# Scottish independence: the view of psychiatry from Edinburgh

**DOI:** 10.1192/pb.bp.116.054965

**Published:** 2017-08

**Authors:** Julia Bland

**Figure:**
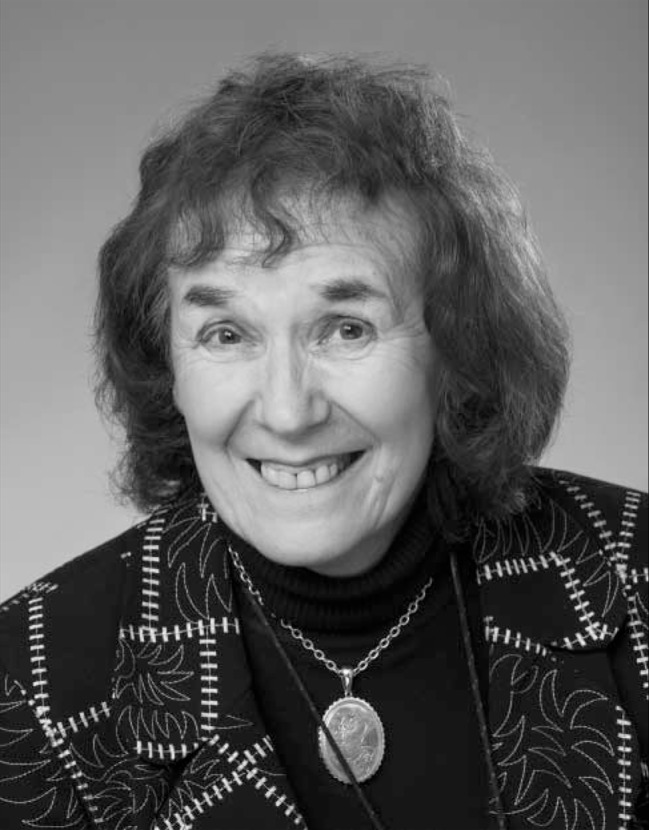


As a southerner and medical psychotherapist, it was with some trepidation that I ‘Ubered’ to Professor Eve Johnstone's house in Edinburgh, which boasts over a hundred roses in her immaculate garden. After all, I was going to meet the woman whose research work had wrenched the pendulum radically in the direction of biological psychiatry, with her landmark *Lancet* paper^1^ showing anatomical differences on CT scans between the brains of those with and without schizophrenia. I feared that she ate psychotherapists for breakfast, although she had sounded kind and friendly on the telephone. My anxiety was misplaced, since the person who emerged was a woman of fierce intelligence, with rigorous, idealistic, uncompromising and wholly admirable moral standards; compassionate and committed to patients and science, without any personal vanity, unless you include intellectual certainty.

Professor Johnstone is not a postmodern relativist: her life has indeed been a life scientific. For her this is seamless: the scientific truth is what leads to effective and therefore optimal treatment. She has never given interviews before, owing to a self-deprecating Scottish horror of self-publicity, but agreed to speak now as a retired professor.

Eve Cordelia Johnstone, ‘Scottish neuroscientist’, is the eighth generation of Glaswegian Presbyterian doctors, but the first woman doctor in her family. She laughed gently at my southern ignorance in asking about Catholicism in Glasgow: of course the Catholics were often of Irish origin, poor, poorly educated and discriminated against. No chance of eight generations of doctors then. (In fact, her father was a dentist because his serious deafness made medicine impossible.)

She remembered vividly the poverty and overcrowding in Glasgow, the worst in the UK at the time. One of her first patients on medical take was an exhausted woman presenting with open tuberculosis, a urinary tract infection and a haemoglobin of 8, who arrived late at night, after three jobs that day: scrubbing steps, cleaning offices, then washing dishes. ‘Had she ever been to the hospital before?’ I ask. ‘Yes, to be sterilised after having 13 children.’ The woman was 35 and looked 20 years older. Infant mortality was 28/1000 in Glasgow at this time, compared with about 6/1000 now.

Born in 1944, the young Eve was sent to an academic private Presbyterian girls' school and was an obvious high flyer. This remained the case: she qualified in medicine in Glasgow in 1967. As a woman medical student, she was one of a 20% maximum quota. Most of her female contemporaries ended up doing a bit of part-time practice rather than working ‘in a serious capacity’, as she put it.

As a clinical researcher who looked after patients with scrupulous care, she is proud of having been supported by the Medical Research Council (MRC) continuously for 33 years, which is almost unprecedented. She did research at Northwick Park in Harrow from 1974 until 1989, and was Professor and Head of Department of Psychiatry at the University of Edinburgh from 1989 until 2010. She has a reputation in Edinburgh for supporting younger psychiatrists, although she has no time for slacking (or striking, as a doctor): ‘I worked 12 hours a day, it was what I expected’.

## The struggle for schizophrenia

In 2016 it may be hard to remember the climate in which the 1976 *Lancet* paper landed.^1^ Debate on the origin and meaning of mental illness was in the international public domain. The flamboyant R. D. Laing, a fellow Scot, was describing psychosis as a sane response to an insane society, chiming perfectly with the other social and sexual revolutions in progress.^2^ Thomas Szasz published *The Myth of Mental Illness* in the same year, depicting psychiatrists (and all other doctors) as agents of social control.^3^ In 1975 Miloš Forman's *One Flew over the Cuckoo's Nest* portrayed the excesses of medicalisation, incarceration and psychosurgery.^4^ A famous study emerged in the USA in 1973, showing how journalists going to the emergency room complaining of ‘hearing’ three words in their heads attracted the diagnosis of schizophrenia.^5^ This massively discredited conventional psychiatric diagnosis. In the UK, David Cooper published *The Death of the Family* (1971), describing the family as the crucible of mental illness, and coined the term ‘anti-psychiatry’.^6^ The now discredited and mother-blaming notion of the ‘schizophrenogenic mother’ was widely held.

So just imagine the hostile reaction that a paper showing anatomical changes in schizophrenia would receive. As Professor Johnstone remembers: ‘There was all this anti-psychiatry stuff […] People were saying schizophrenia didn't exist […] I had to prove my opponents wrong.’ As she reminisces today, it is not difficult to detect her contrarian relish for the battle: Professor Johnstone had no time for these theories. ‘There were 200 000 people in hospital with schizophrenia [… ] what did they think they were there for?’ she asks incredulously. It was also the time that John Wing had written about the phenomenon of institutionalisation,^7,8^ although she points out ‘he never claimed that the institutions were the cause of the illness’.

In her own mind, Professor Johnstone was crystal clear:

‘I felt it had to be that this was a disease, but I couldn't prove it […] and then the non-invasive method of CT scanning came in. I was lucky. Before that people had to do pneumoencephalography (injecting air into the ventricles and X-raying them, resulting in terrible headaches and worse), so there were no controls because of the dangers of the technique. The papers were all in Japanese or German.’

This is a chilling moment: we are talking the Third Reich. ‘It sounds awful to admit’, she says ‘but the best papers [in support of the notion of schizophrenia as a disease originated in] the Third Reich. The Nazis wanted proof that schizophrenia was an inherited degenerative condition.’ When the 1976 study was published, even other neuroscientists objected, suggesting that the anatomical change could be secondary to drug treatment or encephalitis. ‘But I knew something was wrong with these people [… ] When I was 21, I saw a 21-year-old woman, terribly distressed by paranoid delusions, who had been working in a bank 3 weeks earlier. It was ridiculous to say this was due to imperfect interpersonal reactions at home [… ] just stupid.’

With iron determination, she then went on to refute all the challenges to the original paper with a study of 600 people with schizophrenia in Shenley hospital.^9^ They were followed up until death, and their brains at autopsy showed larger ventricles and smaller brains. However, there was no gliosis, suggesting that the pathology may be neurodevelopmental. Professor Johnstone, for all her modesty, cannot suppress an element of glee in having pipped to the post her American research competitors. Daniel Weinberger, backed by the enormous funds of the National Institute for Mental Health, confirmed her findings 2.5 years later.^10^ Not shirking controversy, she then ran a placebo-controlled trial of electroconvulsive therapy (ECT), which demonstrated ECT to be effective, albeit only for about 8 weeks, particularly in those depressed patients who experienced delusions and had intellectual disability.

Her next project, the Edinburgh High Risk Study,^11^ was a prospective study of 16- to 22-year-olds, carefully comparing the incidence of developing schizophrenia in a cohort of people whose relatives had schizophrenia, the controls having no family history. Twenty-one of the high-risk group who then developed schizophrenia in fact had some anatomical differences in the brain when they were still well. The changes include smaller amygdala and hippocampus and hyperfolded gyrification. Of course, this finding raises a host of ethical issues in relation to premorbid diagnosis and its implications.

I asked Professor Johnstone what she thought of the recovery model, and she was sceptical:

‘Seems to me a bit of a semantic issue […] ifs a dreadful illness, very hard for families, specially if you knew the people before they became ill […] and people don't want to say how bad it's going to be. I don't think the [Royal College of Psychiatrists] has emphasised psychopharmacology enough. We need much more individually tailored treatments, taking account of distinct side-effect profiles, not general algorithms. Unfortunately, it's not true that the drugs we have now are vastly better, although it is better to have a wider range.’

She sees non-pharmacological treatment as ‘a bit better than nothing, if [the patient] will engage’, but her scrupulous honesty demands that we acknowledge that ‘a terrible thing has happened to them.’ Talking to her it was clear that she personally provided intensive clinical care to her patients and their families, and we could easily agree that continuity of care is crucial and dangerously eroded in many overstretched contemporary psychiatric services. She is unconvinced of the benefits of the newer antipsychotics: ‘It's true the side-effects are different, but they hammer weight on to you, which is very distressing for young people.’ As for clozapine, the benefits have been ‘greatly exaggerated’.

Her academic success has been recognised. She received a CBE in 2002 for services to medicine, and a Lifetime Achievement Award of the European Psychiatric Association in 2009. She also had many senior roles in the MRC, including the chairing of the highly publicised inquiry into the disastrous linking of the MMR (measles, mumps and rubella) vaccine and autism. With the distressed parents of autistic children leading a class action against the government on the basis of the Wakefield paper,^12^ she was truly in the eye of the storm. ‘I had to carry the parents with me.’ The disgraced Wakefield ‘got what he deserved’, she says.

In retirement Professor Johnstone has remained actively interested in the link between schizophrenia and cognitive impairment. She reminds me that there is a 3% point prevalence of schizophrenia in people with mild intellectual disability, three times higher than in the general population. The focus of understanding is in the area of overlap of schizotypal cognitions, molecular genetics and anomalies of brain structure, where ‘we are seeing a final common pathway that leads to schizophrenia.’ The large data-sets she has in this area fit well with the work of the Patrick Wild Centre, a charitable trust in Edinburgh set up for research into autism, fragile X syndrome and intellectual disability. Politically savvy and persuasive, she raised the funds for the centre, named after a person with severe autism.

## Future hopes

In terms of the future, she told me about some hopeful developments in Edinburgh, which have potential for a personalised preventive psychiatry. Starting with a simple skin biopsy fibroblast, stem cells and then neurones can be grown, giving an opportunity to ‘develop drug treatments which basically work on an individual patient's brain which has been developed in a dish’, as Professor Stephen Lawrie, her successor at the University of Edinburgh, put it. Roll on the day.
